# MicroRNA-124 Alleviates Retinal Vasoregression via Regulating Microglial Polarization

**DOI:** 10.3390/ijms222011068

**Published:** 2021-10-14

**Authors:** Ying Chen, Jihong Lin, Andrea Schlotterer, Luke Kurowski, Sigrid Hoffmann, Seddik Hammad, Steven Dooley, Malte Buchholz, Jiong Hu, Ingrid Fleming, Hans-Peter Hammes

**Affiliations:** 1 5th Medical Department, Medical Faculty Mannheim, University of Heidelberg, D-68167 Mannheim, Germany; Ying.Chen@medma.uni-heidelberg.de (Y.C.); Jihong.Lin@medma.uni-heidelberg.de (J.L.); Andrea.Schlotterer@medma.uni-heidelberg.de (A.S.); Luke.Kurowski@medma.uni-heidelberg.de (L.K.); 2 Center of Medical Research, Medical Faculty Mannheim, University of Heidelberg, D-68167 Mannheim, Germany; Sigrid.Hoffmann@medma.uni-heidelberg.de; 3 Molecular Hepatology Section, Department of Medicine II, Medical Faculty Mannheim, University of Heidelberg, D-68167 Mannheim, Germany; Seddik.Hammad@medma.uni-heidelberg.de (S.H.); Steven.Dooley@medma.uni-heidelberg.de (S.D.); 4 Department of Gastroenterology and Endocrinology, University Hospital, Philipps-University Marburg, Hans-Meerwein-Str. 3, D-35043 Marburg, Germany; malte.buchholz@staff.uni-marburg.de; 5 Institute for Vascular Signalling, Center for Molecular Medicine, Goethe University, D-60590 Frankfurt, Germany; Hu@vrc.uni-frankfurt.de (J.H.); Fleming@em.uni-frankfurt.de (I.F.)

**Keywords:** miR-124, microglia, polarization, vasoregression

## Abstract

Microglial activation is implicated in retinal vasoregression of the neurodegenerative ciliopathy-associated disease rat model (i.e., the polycystic kidney disease (PKD) model). microRNA can regulate microglial activation and vascular function, but the effect of microRNA-124 (miR-124) on retinal vasoregression remains unclear. Transgenic PKD and wild-type Sprague Dawley (SD) rats received miR-124 at 8 and 10 weeks of age intravitreally. Retinal glia activation was assessed by immunofluorescent staining and in situ hybridization. Vasoregression and neuroretinal function were evaluated by quantitative retinal morphometry and electroretinography (ERG), respectively. Microglial polarization was determined by immunocytochemistry and qRT-PCR. Microglial motility was examined via transwell migration assays, wound healing assays, and single-cell tracking. Our data showed that miR-124 inhibited glial activation and improved vasoregession, as evidenced by the reduced pericyte loss and decreased acellular capillary formation. In addition, miR-124 improved neuroretinal function. miR-124 shifted microglial polarization in the PKD retina from the pro-inflammatory M1 phenotype to the anti-inflammatory M2 phenotype by suppressing TNF-α, IL-1β, CCL2, CCL3, MHC-II, and IFN-γ and upregulating Arg1 and IL-10. miR-124 also decreased microglial motility in the migration assays. The transcriptional factor of C/EBP-α-PU.1 signaling, suppressed by miR-124 both in vivo (PKD retina) and in vitro (microglial cells), could serve as a key regulator in microglial activation and polarization. Our data illustrate that miR-124 regulates microglial activation and polarization. miR-124 inhibits pericyte loss and thereby alleviates vasoregression and ameliorates neurovascular function.

## 1. Introduction

Vasoregression is the primary process of various retinal disorders such as retinal degeneration and diabetic retinopathy (DR). These disorders are characterized by pericyte loss and acellular capillary formation. Microglial activation contributes to vasoregression and neurodegenerative retinopathy [1]. Microglia, Müller cells, and astrocytes comprise the three types of glia cells in the mammalian retina [2,3]. Glial cells play an important role in cross-communication between neurons and vascular cells (collectively known as neurovascular units (NVUs)), especially in retinal homeostasis, inflammation, and neurodegeneration [4]. In the normal retina, the microglia is quiescent and distributes in the inner plexiform layer with ramified morphology. When exposed to various pathological insults, microglia become activated, change to amoeboid forms, migrate to the site of injury, secrete anti-inflammatory cytokines such as interleukin-10 (IL-10) and Arginase-1 (Arg1), and facilitate the resolution of inflammation and the return of the tissue to homeostasis [5,6]. However, in chronic pathological conditions such as diabetic retinopathy, microglia release excessive pro-inflammatory chemokines, including C-C motif chemokine ligand 2 (CCL2) and CCL3 or cytokines such as tumor necrosis factor-α (TNF-α), interleukin-1β (IL-1β), and interferon gamma (IFN-γ), which promote disease progression [4,6,7]. Abnormal microglia activation has also been associated with retinal degenerative diseases, including age-related macular degeneration and hereditary retinopathies [2,4]. The effective modulation of microglial polarization to the anti-inflammatory state could inhibit neurodegenerative disease progression [8,9]. Modulating the state of microglial activation and its response to the inflammatory environment might be an approach to prevent neurovascular change resulting in retinal vasoregression [10,11].

Transgenic PKD rats overexpress the human polycystin-2 gene in retinal photoreceptors, among other tissues [12,13]. They are characterized by abnormal cellular cilia and retinal degenerative phenotypes with severe photoreceptor degeneration, vasoregression, and microglial activation [14]. Microglial activation promotes the expression of immune cytokine CD74, a receptor for the macrophage migration inhibitory factor [15] and the invariant chain of the class II major histocompatibility complex (MHC-II) [16]. CD74 is considered a marker of microglial activation, and it is closely correlated with vasoregression in PKD rats [17]. As a spontaneous neurodegeneration model very similar to human ciliopathy, the PKD rat has been identified as a useful model to study the relationship between neurodegeneration and vasoregression [14,17]. Ciliopathy is a group of human genetic disorders exhibiting major retinal degeneration (rod cone dystrophy and photophobia), sensorineural hearing loss, obesity, and insulin resistance [18].

Given the regulatory role of miRNAs in neurodegeneration, the impact of specific miRNAs needs clarification. miRNAs are involved in many biological processes, while altered expression of miRNAs is associated with the development of many human diseases [19]. miRNA-124 (miR-124) is one of the most abundant miRNAs in the CNS and retina. It regulates physiological neurogenesis and neuronal development [20,21,22]. The regulatory role of miR-124 in microglial activation has been studied in several disease models of inflammation, and its application was suggested as a promising therapeutic approach [23]. The delivery of miR-124 into a rat model of a spinal cord injury reduced microglia activation and inflammatory cytokine production [24]. Supplementation of miR-124 inhibits the pro-inflammatory activity of Müller cells in a photo-oxidative damage mouse model [25]. miR-124 promotes microglia quiescence in the CNS of an experimental autoimmune encephalomyelitis (EAE) mouse model and represses EAE disease development. In this model, the transcriptional factor protein binding U box (PU.1) and its upstream regulator, CCAAT/enhancer-binding protein-α (C/EBP-α), are involved in miR-124-mediated microglia regulation [26].

However, the effect of miR-124 on retinal vasoregression remains unclear. Our aim was to clarify whether miR-124 regulates retinal microglia activation and subsequently influences retinal vasoregression. We found that miR-124 suppressed retinal microglial activation and polarized microglia to the anti-inflammatory phenotype in the PKD rat. The extensive vasoregression of the PKD retinae and the neuroretinal function were improved by miR-124 introduction. The C/EBP-α/PU.1 signaling pathway was involved in miR-124-mediated retinal microglial activation and polarization.

## 2. Results

### 2.1. miR-124 Normalizes Müller Glial Activation in the PKD Retina

**Figure 1:**
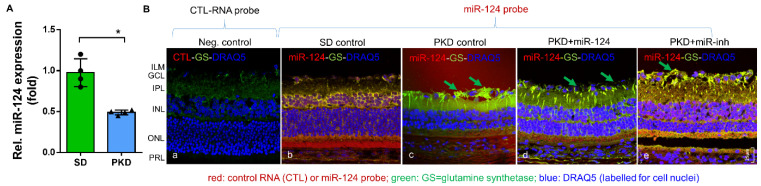
miR-124 expression was reduced in the retinae of PKD rats and inactivated the Müller glia. (**A**) Relative expression of miR-124 in the retinae of SD and PKD rats measured by RT-qPCR using hsp-miR-124-3p specific primers. Data are normalized to the expression of U6 snRNA, exhibited as means ± SD, *n* = 4. The *p* value determined by a Student’s *t* test was * *p* < 0.05. (**B**) ISH combined with fluorescent immunohistochemistry. A control RNA probe (**a**) or miR-124 probe (**b**–**e**) was used to detect paraffin-embedded retinal vertical sections from the SD (**a**,**b**) and PKD rats with (**d**) or without miR-124 (**c**) or miR-inh (**e**) injection. The miR-124 probe was labeled with donkey anti-sheep Alexa Fluor 555 (red), the glutamate synthetase (GS) was labeled with chicken anti-rabbit Alexa Fluor 488 (green), and the nuclei were labeled with DRAQ5^TM^ (blue). A section from the SD retina probed with the control miRNA probe (CTL-RNA probe) served as a negative control (**a**). The images were taken with Leica confocal microscope TCS SP8, and scale bar shown in B, e = 25 µm.

To assess miR-124 change in the PKD rat, we performed quantitative RT-PCR. The PKD rat retinae expressed 50% less miR-124 than the SD rats (Figure 1A). To ascertain the localization of miR-124 and to determine whether exogenous miR-124 could influence Müller glial activation, we introduced an miR-124 mimic into the PKD retina and performed in situ hybridization (ISH) combined with immunofluorescent staining. Neither the hybridization signal nor the immunofluorescence signal of glutamine synthetase (GS) in the negative control group of the SD retinae were detectable (Figure 1(Ba)). ISH confirmed the RT-qPCR result that the miR-124 expression was reduced in the PKD retina (Figure 1(Bc)) compared with the expression in the normal SD retina (Figure 1(Bb)). miR-124 was expressed in all layers of the SD retina, predominantly in the photoreceptor layer (PRL) but also in the ganglion cell layer (GCL) and the inner nuclear layer (INL) (Figure 1(Bb)), yet its expression was strikingly decreased in all layers of retinal neurons in the PKD rats (Figure 1(Bc)). Additionally, miR-124 was distributed across the entire retina (Figure 1(Bd,e)), consistent with the Müller cell processes spanning across all retinal layers. The immunofluorescent signal of the Müller glial GS was increased, and the Müller cells showed gliosis in the PKD retinae (arrows in Figure 1(Bc)), while the exogenous miR-124 inhibited GS expression and indicated a reduction of Müller glial activation (arrows in Figure 1(Bd)) [14,17]. The miR-124 inhibitor did not affect Müller glial activation (Figure 1(Be)), indicating that Müller glial activation was inhibited by miR-124.

### 2.2. miR-124 Decreases the Activation State of Microglia in PKD Retinae

**Figure 2:**
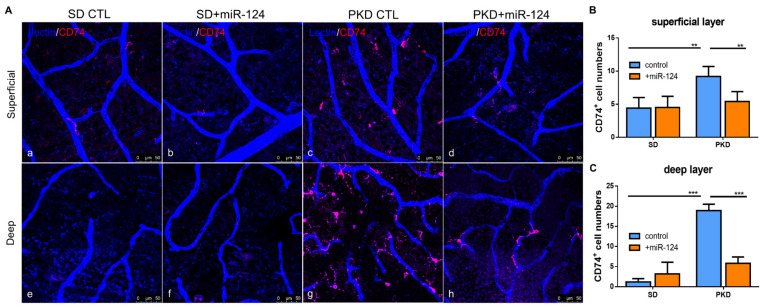
Microglial distribution and activation in SD and PKD retinae, with whole-mount immunofluorescent staining of CD74 in retinae from SD (**a**,**b**,**e**,**f**) and PKD (**c**,**d**,**g**,**h**) rats treated with either control miRNA (CTL) (**a**,**c**,**e**,**g**) or an miR-124 mimic (miR-124) (**b**,**d**,**f**,**h**). Microglia were labeled with CD74 (red). Retinal vessels were labeled with iso-lectin B4 (blue). (**A**) Representative images of microglial activation (CD74^+^) in the superficial layer (upper panel) and in the deep layer (lower panel). (**B**) Quantification of the CD74^+^ microglia in the superficial layer (upper panel A). (**C**) Quantification of the CD74^+^ microglia in the deep layer (lower panel A). The images were taken with a Leica confocal microscope TCS SP8 with scale bars = 50 µm. Data (**B**,**C**) are exhibited as means ± SD, *n* = 5. The *p* values were determined by two-way ANOVA with Tukey’s multiple comparisons test were ** *p* < 0.01 and *** *p* < 0.001.

Neurodegeneration is characterized by a strong microglial activation. To assess whether miR-124 can modify microglial behavior, the expression of the microglial activation marker CD74 was analyzed in the whole-mount retinae of SD and PKD rats. Microglia are abundant and activated in the entire retina of PKD rats (both in the superficial and deep layer) (Figure 2(Ac,g)) in comparison with the levels in the SD retina (Figure 2(Aa,e)). The delivery of the miR-124 mimic into the PKD retina attenuated microglial activation both in the superficial and the deep layer (Figure 2(Ad,h)). In the deep layer, miR-124 reduced 80% of the activated microglial cell numbers while reducing them 50% in the superficial layer (Figure 2B,C) compared with the PKD rat without an miR-124 injection. miR-124 did not influence the microglia activation in the SD rats (Figure 2(Aa,b,e,f)).

### 2.3. miR-124 Alleviates Vasoregression of the PKD Retina

**Figure 3:**
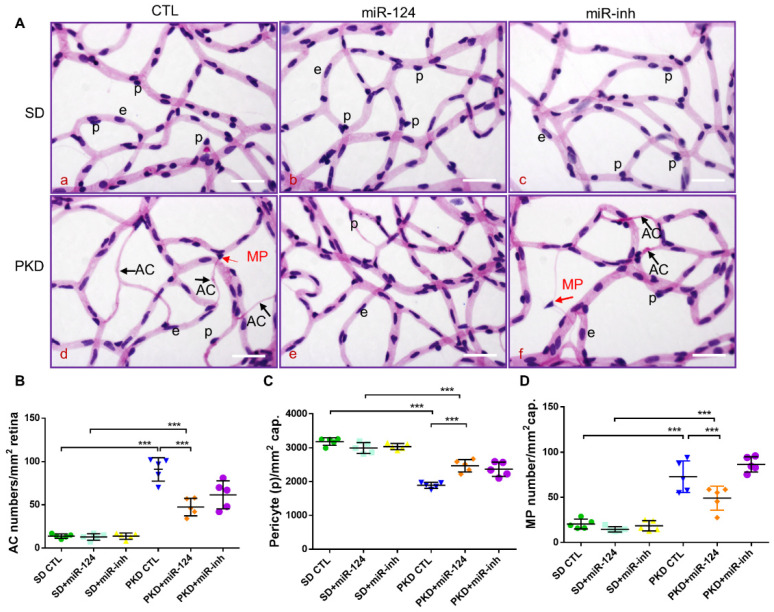
miR-124 ameliorates pericyte loss and reduces vasoregression in PKD retinae. Retinal morphometry was measured in SD and PKD rats treated with or without miR-124. Two-month-old SD and PKD rats were treated with 25 pmol of control microRNA (CTL), an miR-124 mimic, or miR-124 inhibitor (miR-inh) for 4 weeks. (**A**) Representative images of PAS and hematoxylin-stained retinal vasculature from retinal digest preparation taken by an Olympus BX51 microscope. Black arrows indicate acellular capillaries (ACs), red arrows indicate migrating pericytes (MPs), p = pericyte, e = endothelial cell, and scale bars = 50 µm. (**B**–**D**) Quantification of acellular capillaries (number of ACs/mm^2^ retinal area) (**B**); quantification of pericytes (number of pericytes/mm^2^ retinal area) (**C**); and quantification of migrating pericytes (number of MP/mm^2^ retinal area) (**D**) were analyzed using Cell^F^ software from Olympus. (**B**–**D**) *n* = 5 and *** *p* < 0.001 (one-way ANOVA with Tukey’s multiple comparisons test).

To determine whether exogenous miR-124 administrated intravitreally could rescue retinal vasoregression, retinal digest preparations of SD and PKD rats treated with an miR-124 mimic, control miRNA (CTL-miR), or miR-124 inhibitor (miR-inh) were analyzed using morphometry. As expected, PKD retinae displayed increased formations of acellular capillaries (ACs) (Figure 3(Ad),B). miR-124 treatment did not affect the low number of ACs in the SD rats (Figure 3(Ab),B). However, it significantly reduced the numbers of AC formation in the PKD retinae (Figure 3(Ae),B). In contrast to miR-124, the inhibitor (miR-inh) did not affect AC formation in both the SD and PKD rats (Figure 3(Ac,f),B). Pericyte numbers were significantly reduced in the retinae from the PKD rats (Figure 3(Aa,d),C). miR-124 also increased the pericyte numbers in the PKD rats (Figure 3(Ab,e),C). Pericyte migration, often found in damaged retinae, was also increased in the PKD retinae and inhibited by miR-124 addition (Figure 3(Ab,e),D). The miRNA inhibitor did not affect pericyte migration (Figure 3(Ac,f),D), which was consistent with the persistent AC formation and pericyte loss under in vivo treatment (Figure 3B–D).

### 2.4. miR-124 Improves Retinal Function in Neurodegeneration

**Figure 4:**
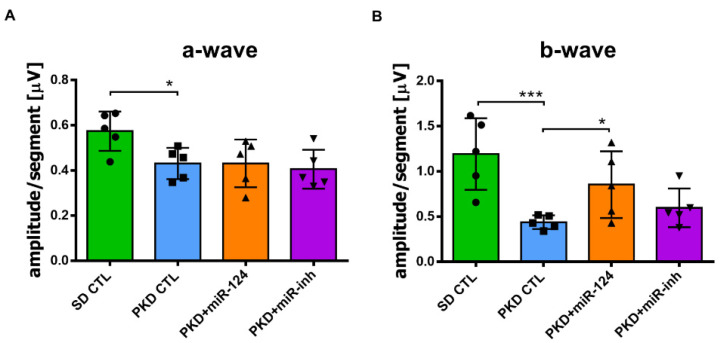
miR-124 ameliorated neuroretinal function in the PKD rats. The neuroretinal function was measured via electroretinography (ERG) in the SD and PKD rats. SD and PKD rats were intravitreally administrated with 25 pmol of the control miRNA (CTL), miR-124 mimic, or miR-124 inhibitor (miR-inh) at week 8 and week 10, respectively. ERG was performed at week 12. Data are presented as means ± SD, *n* = 5. The *p* values were determined by one-way ANOVA with Tukey’s multiple comparisons test, where * *p* < 0.05 and *** *p* < 0.001. (**A**) Represented a-wave amplitudes in ERG. (**B**) Represented b-wave amplitudes in ERG.

Given that miR-124 mitigated PKD Müller glial activation and retinal vasoregression, which are the successors of neurodegeneration, we assessed the effect of miR-124 on neuroretinal function using electroretinography (ERG). The a-wave amplitude reflects the function of the photoreceptors, while the b-wave amplitude represents the function of the bipolar cells and Müller cells. In the PKD retina, both the functions of photoreceptors (Figure 4A) and of bipolar cells and Müller cells (Figure 4B) were reduced compared with the SD retina. Photoreceptor function of the PKD rat was not improved by miR-124 mimic administration (Figure 4A). However, after 4 weeks of treatment with the miR-124 mimic, the response to light of the inner retinal neurons of the PKD rat was significantly recovered to the level present in the SD control (Figure 4B). Altogether, miR-124 not only regulated Müller glia activation and microglial activity but also rescued vasoregression and neuroretinal functions of the PKD rat.

### 2.5. miR-124 Inhibits the Migratory Capability of Microglia

**Figure 5:**
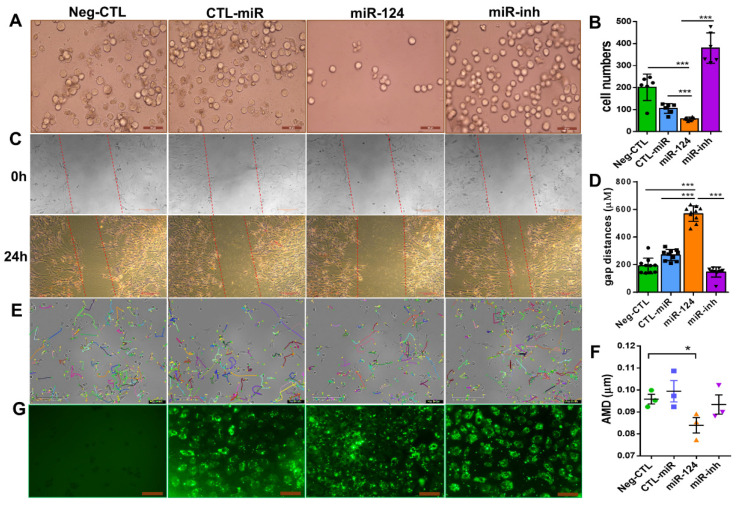
miR-124 reduced microglial cell motility. BV2 microglial cells (**A**,**E**,**G**) and rat primary microglial cells (**C**) were transfected with Lipofectamine 2000 Transfection Reagent as a negative control (Neg-CTL), control microRNA (CTL-miR), microRNA-124 (miR-124), and miR-124 inhibitor (miR-inh) for 24 h (**A**,**C**,**E**,**G**). (**A**) Images of transwell migration assays taken with a Zeiss Axio Observer Z1 microscope, where the scale bars = 50 µm. (**B**) Quantification showed that the migrating cells moved downward well with the serum-free medium after 24 h. (**C**) Images of wound healing assays with rat primary microglial cells at time point 0 h (upper panel) and end time point 24 h (low panel), with scale bars = 200 µm. (**D**) Quantification of the gap sizes from the 24 h-panel of (**C**). (**E**) Tracking of single BV2 cell movement. After 24 h of transfection, cells were applied to live cell imaging with an Incucyte S3 phase contrast microscope. Images of three fields per well were taken at 30-min intervals, and cell tracking was monitored for 10 h. Tracks of individual cells are shown in different colors, where the scale bars = 200 µm. (**F**) Quantification of the average migration distance (AMD in µm) of a single cell was analyzed with the TimeLapse Analyzer (TLA). Three fields each of 100 cells were analyzed, where *n* = 3 and * *p* < 0.05 (one-way ANOVA with Tukey’s multiple comparisons test). (**G**) Transfection evidence of BV2 cells with FAM-conjugated control miRNA, an miR-124 mimic, or miR-124 inhibitor observed using a Zeiss Axio Observer Z1 phase-contrast fluorescent microscope, with scale bars = 100 µm. (**B**) *n* = 5. (**D**) *n* = 10 and *** *p* < 0.001 (one-way ANOVA with Tukey’s multiple comparisons test).

Given that migration is a key feature during microglial surveillance and an important characteristic of microglial activation [4,27], we investigated the effect of miR-124 on the migratory capability of mouse BV2 microglia and rat primary microglia. Via a transwell migration assay, 24 h after transfection, the number of BV2 cells that migrated to the lower transwell chamber was effectively inhibited by 50% in the group transfected with the miR-124 mimic compared with the control miRNA (CTL-miR) (Figure 5A,B). A wound healing assay (WHA), which is used to measure the cell moving speed, confirmed our findings. Twenty-four hours after transfection, the miR-124 mimic transfected rat primary microglial cells traveled much slower than the CTL-miRNA or miR-124 inhibitor transfected control cells (Figure 5C,D). Similar results for the WHA were observed in mouse microglia BV2 cells (data not shown). The single-cell mobility was assessed by live cell imaging techniques, accomplished with an Incucyte S3 phase contrast system and analyzed with the TimeLapse Analyzer (TLA) program. The average migrating distance (AMD, in µm) of miR-124 transfected BV2 cells was significantly shorter (*p* < 0.05) than the non-transfected control group (Neg-CTL) and shorter than the CTL-miRNA and miR-124 inhibitor groups (Figure 5E,F), indicating that microglial motility was inhibited by miR-124’s introduction.

To evaluate the transfection efficiency and to position miR-124 in the microglial cells, an FAM-labeled miR-124 mimic, control miRNA (CTL-miR), or miRNA inhibitor (miR-inh) were transfected into the microglial cells. The 24-h transfection efficiency in each experimental group of BV2 cells was similar in green fluorescence intensity (Figure 5G).

### 2.6. miR-124 Modulates the Polarization of Microglia to an Anti-Inflammatory Phenotype

**Figure 6:**
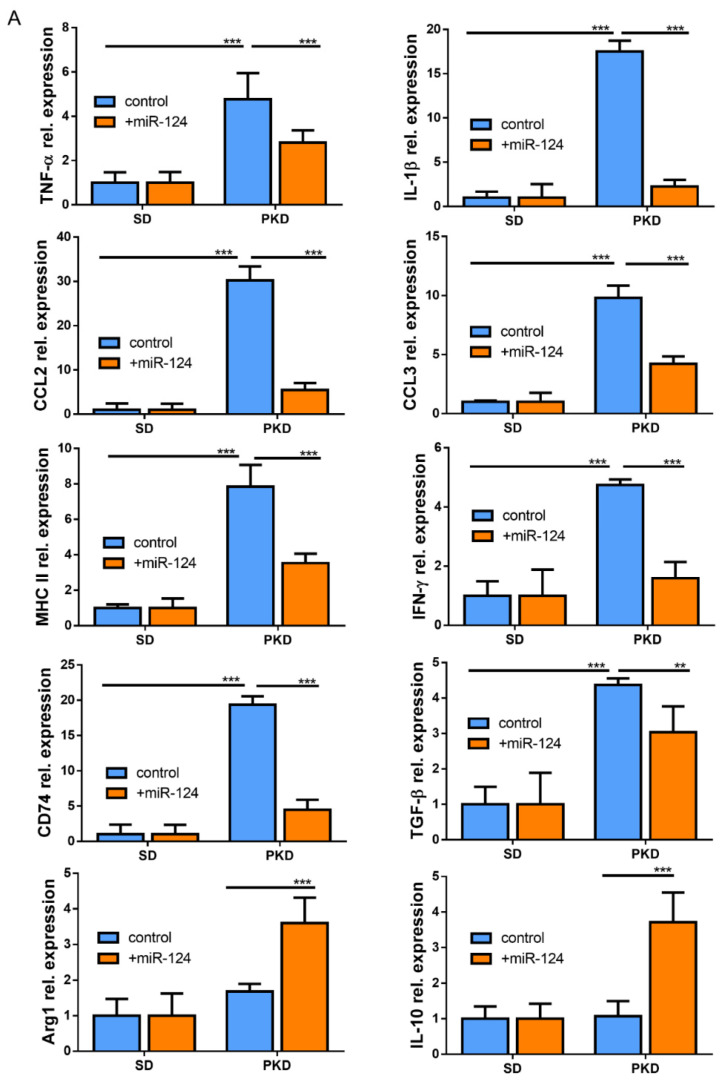

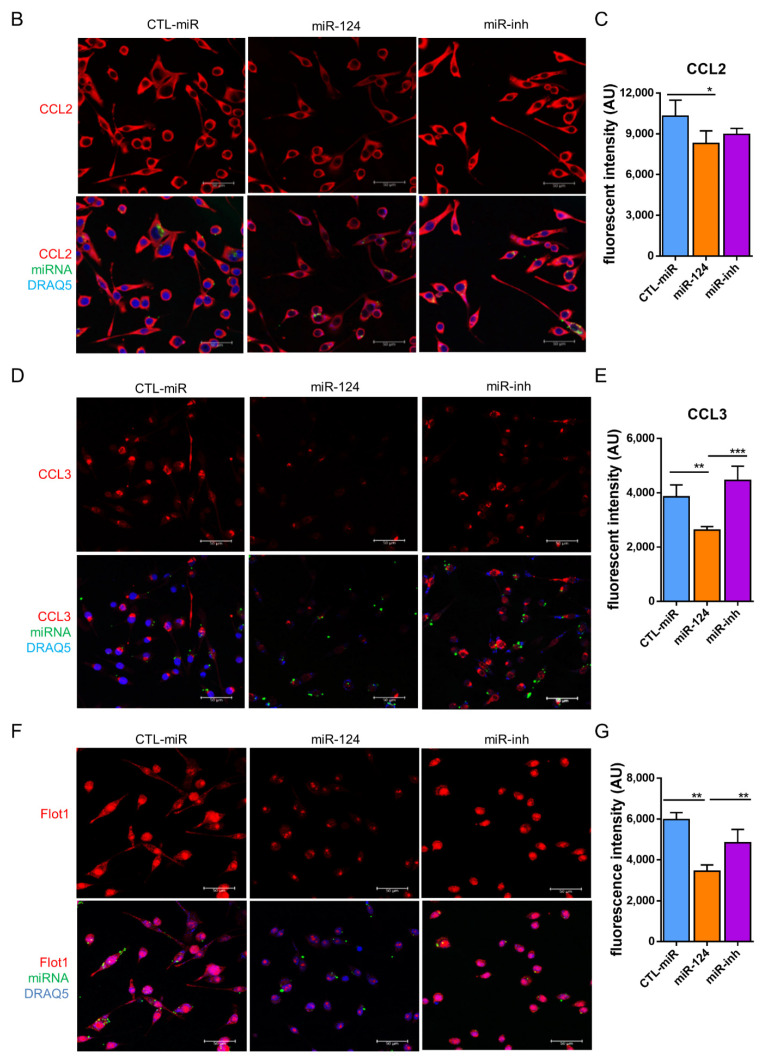
miR-124 reduced expression of pro-inflammatory cytokines in the PKD rat. (**A**) Quantitative M1 and M2 gene expressions in SD and PKD rats treated with or without the miR-124 mimic. RT-qPCR was performed to evaluate the genes specially expressed in the M1 state (TNF-α, IL-1β, IFN-γ, CCL2, CCL3, MHC-II, CD74, and TGF-β) and M2 state (Arg1 and IL-10). The expression of the house-keeping gene, rat Gapdh, was used as a control. Relative gene expression (fold versus Gapdh) was calculated using the ∆∆CT method. (**B**,**D**,**F**) Representative images of fluorescent immunocytochemistry (ICC) of CCL2 (**B**), CCL3 (**D**), and Flot1 (**F**) in BV2 cells. BV2 cells were transfected with FAM-labeled miR-124 or an miR-124 inhibitor (miR-inh) (green) for 24 h. Antibodies of CCL2, CCL3, and Flot1 were labeled with Alexa Fluor 555 (red), and the nuclei were labeled with DRAQ5^TM^ (blue). Images were taken using a Leica confocal microscope TCS SP8, with scale bars = 50 µm. (**C**,**E**,**G**) Quantification of CCL2 (**C**), CCL3 (**E**), and Flot1 (**G**) expressions from ICC fluorescence intensity using Image J software. Data are shown in arbitrary units (AU). (**A**,**C**,**E**,**G**) *n* = 5, * *p* < 0.05, ** *p* < 0.01, and *** *p* < 0.001 (two-way ANOVA with Tukey’s multiple comparisons test).

To identify whether miR-124 could influence the microglia response to inflammation, the expressions of pro-inflammatory M1 and anti-inflammatory M2 mediators or specific secreted cytokines were evaluated by RT-qPCR in the retinae of SD and PKD rats treated with the miR-124 mimic for 4 weeks. In the PKD retinae, miR-124 reduced the expression of pro-inflammatory cytokines (TNF-α, IL-1β, and IFN-γ), chemokines (CCL2 and CCL3), MHC-II, CD74, and TGF-β1, whereas it promoted the expression of the M2 marker Arg1 and anti-inflammatory cytokine IL-10 (Figure 6A). The fact was that the expression of pro-inflammatory chemokines CCL2 and CCL3 was suppressed by miR-124 was also confirmed at the protein level by ICC (Figure 6 B–E). These data supported the capability of miR-124 to shift microglia to the anti-inflammatory phenotype.

Flotillin 1 (Flot1) is a target of miR-124 and an important protein in lipid raft formation [28]. To further investigate the mechanism of miR-124 on the anti-inflammatory phenotype of microglia, we examined the expression of Flot1 in BV2 microglial cells using ICC. The expression of Flot1 was inhibited by about 40% in miR-124-transfected BV2 cells compared with the control miRNA or miR-124 inhibitor treated groups (Figure 6F,G). We concluded that miR-124-induced Flot1 reduction might have interfered with lipid raft-mediated inflammatory signal transduction, which inhibited pro-inflammatory signals and promoted anti-inflammatory signals to the microglia.

### 2.7. miR-124 Downregulates the Transcription Factors C/EBP-α/PU.1

**Figure 7:**
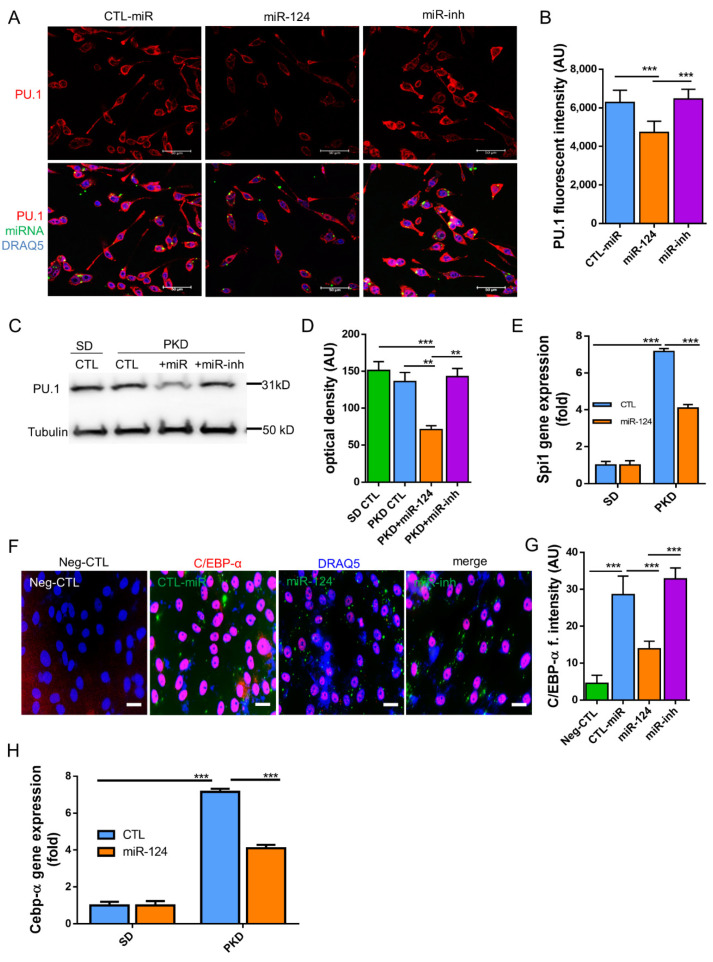
miR-124 regulated the expression of PU.1 and C/EBP-α in microglial cells and in PKD retinae. (**A**,**F**) Immunocytochemistry (ICC) of PU.1 and C/EBP-α expression in BV2 cells. BV2 cells were transfected with an miR-124 mimic (miR-124), control miRNA (CTL-miR), or miR-124 inhibitor (miR-inh). Cells transfected with only Lipofectamine 2000 Transfection Reagent were used as a negative control (Neg-CTL). PU.1 (**A**) and C/EBP-α (**F**) were detected using an Alexa 555-labeled secondary antibody (red), miRNA localization was visualized by FITC (green), and cell nuclei were labeled with DRAQ5^TM^ (blue). Images were taken using a Leica confocal microscope TCS SP8, with scale bars = 50 µm. (**B**,**G**) Quantification of ICC fluorescence from PU.1 (**B)** and C/EBP-α (**G**). (**C**) Western blot detection of PU.1 in the lysates of SD and PKD retinae, where 10 µg of total protein preparation from retina tissue was separated in 4–20% SDS-PAGE gel, and an anti-PU.1 antibody 1:1000 dilution was used for detection. Protein expression of α-Tubulin was used as an internal control. (**D**) Quantification of western blots by optical intensity. Data are represented as means ± SEM. (**E**,**H**) Gene expression of Spi1 (PU.1) and Cebp-α in miR-124 mimic- or control miRNA-treated retinae of SD and PKD rats was evaluated by RT-qPCR. The rat Gapdh gene was used as a housekeeping control. The immunostaining density was quantified with Image J software (**B**,**G**). Data are shown as the mean fluorescent intensity of five images of each condition, where *n* = 5, ** *p* < 0.01, and *** *p* < 0.001 (one-way ANOVA with Tukey’s multiple comparisons test) (**A**–**H**).

Next, we explored the possible mechanisms underlying the above observations. It has been reported that miR-124 promotes microglia quiescence in CNS and downregulates PU.1 in bone marrow-derived macrophages (BMDMs) [26]. The expression of C/EBP-α, a master transcription factor which is a target of miR-124, as well as its downstream transcription factor PU.1 was detected both in vitro (BV2 cells) and in vivo (PKD retinae). PU.1 expression was downregulated in the miR-124 mimic-transfected BV2 cells but not in the control miRNA- or miR-124 inhibitor-transfected groups (*p* < 0.01, Figure 7A,B). To confirm the in vitro experiment, we examined the PU.1 protein expression by western blotting in samples of the SD and PKD retinae. PU.1 suppression was observed in the miR-124 mimic-treated PKD retina by western blot analysis but not in the control miRNA- or miRNA inhibitor-treated PKD or in the control SD retinae (*p* < 0.01, Figure 7C,D). The mRNA of PU.1, known as the Spi1 (Spi-1 Proto-Oncogene) gene, in comparison with the level in the SD retina, was 7.1-fold higher in the PKD retina, which was, however, neutralized to about 56.3% expression in the control miRNA-treated groups through miR-124 mimic introduction (Figure 7E). C/EBP-α expression was reduced in the miR-124-transfected BV2 cells by 50% on the protein level (Figure 7F,G) and by 45% on the mRNA level (Figure 7H). These data indicated that C/EBP-α/PU.1 signaling was involved in the miR-124 dependent regulation of microglial activation and polarization.

## 3. Discussion

In the present study, we investigated the role of miR-124 in retinal vasoregression. Our results demonstrated that the administration of miR-124 in the PKD model of retinal degeneration reduced the activation of Müller glia and microglia as well as microglial motility. The administration of miR-124 downregulated the expression of pro-inflammatory mediators and redirected the response of microglia toward the anti-inflammatory phenotype. The downregulation of the transcription factor PU.1 and its upstream regulator C/EBP-α were associated with microglial modulation. With the replacement of miR-124, pericyte loss and acellular capillary formation were inhibited, and retinal vasoregression was subsequently alleviated.

In comparison with the expression of Müller cells and photoreceptor cells in the SD retina, the level of miR-124 was markedly reduced in the PKD retina. This was likely a consequence of the destroyed photoreceptors in the PKD rats, as the photoreceptor layer is the main source of miR-124, and PKD rats develop severe neurodegeneration between birth and 3 months [14]. We replaced miR-124 in the PKD retina by intravitreal injection. miR-124 expression was inversely correlated with Müller glial and microglial activation. As an important member of the retinal NVU, Müller glia mediate the signal transduction and molecule transport between neurons and vessels mainly through supporting and protecting photoreceptors and neuronal functions [3,29]. Müller glia undergo reactivation and dysfunction under pathological conditions, such as the ciliopathy model of the PKD rat retina [30]. Our results confirmed that the Müller glia underwent gliosis in the PKD rats and their activation was suppressed by miR-124’s introduction. These results indicating that miR-124 inhibited microglial activation and recruitment support our hypothesis that miR-124 is an important modulator of microglia and Müller glia and that it contributes to retinal vasoregression. Upon administration of miR-124, microglial production of M1 markers was reduced, while M2 marker expression was increased in the retina of the PKD rat. This indicates that miR-124 shifts microglia to the anti-inflammatory phenotype. The switch of microglia to the anti-inflammation state may be the important determinant of further disease dynamics. In a spinal cord injury (SCI) mouse model, the viability of microglia was improved when the M1 markers were downregulated and the M2 markers were upregulated [31], supporting the proposed concept. In a mouse model of focal cerebral ischemia, miR-124 shifted the pro-inflammatory microglia and macrophages toward the anti-inflammatory phenotype and induced neuroprotection and functional improvement [32]. Our study demonstrated that miR-124 supports the anti-inflammatory phenotype of microglia in the retina and shifts the retinal microglia toward healing, despite the persistence of the primary insult. The replacement of miR-124 decreased the vasoregression phenotype by increasing the number of pericytes and reducing the extent of acellular capillaries. Müller glial function (b-wave ERG) in PKD rats was rescued by the replacement of miR-124; however, the photoreceptor function (a-wave ERG) could not be recovered. This indicates that miR-124’s mediating of anti-inflammatory microglial function is mainly accomplished through regulating glial activation. The outer retinal degeneration caused mainly by photoreceptor death beginning early in a PKD rat’s life is irreversible. Considering the reciprocal interaction between microglia and Müller glia cells [33,34], as well as by identifying that microglial activation is associated with pericyte loss and capillary formation in the PKD model [17], we speculated that miR-124-regulated vasoregression and Müller glial function was most probably mediated by microglia. When the miR-124 level was sufficient, the microglia were deactivated and polarized to the M2 state. In contrast, when miR-124 was absent in diseased circumstances, the microglia were overactivated and secreted pro-inflammatory molecules, causing vascular cell loss and damaging NVU function.

To elucidate the potentially involved mechanisms by which miR-124 mediated microglial activation and polarization, the transcription factor PU.1 and its upstream regulator C/EBP-α were investigated. C/EBP-α/PU.1 signaling is a key regulator of the miR-124-mediated immune response in the EAE model and CNS inflammation. Overexpression of miR-124 downregulates C/EBP-α/PU.1 expression in macrophages and promotes microglia quiescence [26]. PU.1 is constitutively expressed by peripheral macrophages and brain microglia [35,36]. Its effect in modulating microglia development and activation has been widely explored in neurodegenerative diseases [37,38,39]. Zhou et al. demonstrated that PU.1 is essential for microglial activation in a mouse model of traumatic injury-induced neurodegeneration [39]. When PU.1 expression was downregulated with siRNA, the viability and phagocytotic function of human brain microglia were decreased [37]. In our study, PU.1 was highly expressed in the PKD retina relative to the SD retina. However, after miR-124 replacement, PU.1 expression was reduced in the PKD retina both at the mRNA and protein level. The in vitro study confirmed that the PU.1 expression in microglial cells was decreased by miR-124 transfection. As shown in an in silico analysis, PU.1 is not a direct target of miR-124; rather, it is regulated through the upstream transcription regulator C/EBP-α, a direct binding target of miR-124 [26]. We identified that the expression of C/EBP-α in BV2 cells was also inhibited after the transfection of the miR-124 mimic. PU.1 not only regulates macrophage and microglia activation but is, together with C/EBP-α, also an essential transcription factor for inflammatory polarization [40,41]. In a study on Alzheimer’s disease, downregulation of PU.1 in BV2 cells suppressed the expression of pro-inflammatory genes, such as CCL2 and IL-1β, as well as Aif1 (also known as Iba1), a molecular marker of microglia [37]. In another study with BMDM, miR-150-induced PU.1 downregulation inhibited the expression of pro-inflammatory cytokines and shifted the macrophage polarization toward the M2 state [42]. In a recent study of EAE mice, CNS microglia switched toward the M2 phenotype after BSYS (a traditional Chinese medicine) treatment. Concurrently, the upregulation of miR-124, but reduction of C/EBP-α/PU.1, were observed in vivo [43]. Thus, we assumed that miR-124 supplementation reduced C/EBP-α/PU.1 signaling, which regulated the expression of inflammatory associated genes and subsequently mediated microglial activation and polarization.

In the present study, we found that Flot1 expression was markedly reduced in miR-124 mimic-transfected BV2 cells. Flot1 was identified as a direct target of miR-124 [28]. As a pivotal lipid raft protein, Flot1 is involved in vesicular trafficking and signal transduction [44,45]. Lipid rafts regulate signaling cascades according to the changes of protein–protein interaction by intra- or extracellular stimuli [46]. For instance, upon TNF-α stimulation, excessive TNF receptors (TNFRs) were recruited to lipid rafts and elicited the TNF-α induced inflammation [47]. Dysregulated lipid raft formation and abnormal signaling may lead to the onset of neurodegenerative diseases, including Alzheimer’s disease and Parkinson’s disease [48]. We speculate that TNF-α stimulation was inhibited in the miR-124-treated microglial cells. Concurrently, the disruption of lipid rafts due to Flot1 suppression led to restricted translocation of TNFRs to lipid rafts and consequently to disturbed TNF-α-induced inflammatory responses of the microglia. Flot1 regulates the migration of many cancer cells [28,45,49] and immune cells [50]. Ludwig et al. demonstrated that Flot1 deficiency impairs the migration of neutrophil and the recruitment of immune cells to inflammatory sites [50]. Whether Flot1 downregulation correlates with the migration inhibition of microglia through miR-124 requires further investigation.

Another direct target of miR-124 is the chemokine CCL2 [25]. CCL2 is a potent chemoattractant of microglia and macrophages secreted by Müller cells and thus regulates microglial activation, migration, and recruitment to the position of retinal injury [51,52]. In this study, we identified that the expression of CCL2 was negatively correlated with the level of miR-124 in the PKD retina and BV2 cells, indicating that CCL2 was implicated in the miR-124-regulated microglial activation and inflammatory response. miR-124 inversely regulating CCL2-mediated inflammation has been described in a photo-oxidative damage (PD) mouse model of a degenerating retina [25]. This 5-day PD acute retina degeneration model, combined with our discoveries in the chronic PKD environment, indicates that the replacement of miR-124 modulates the retinal degeneration at least partially with CCL2.

In summary, in the PKD retina, photoreceptor damage causes a reduction of miR-124, resulting in abnormal activation of Müller- and micro-glia and a subsequent increase in vasoregression. After the miR-124 was replaced, Müller glia activation was inhibited, which directed its effector—the microglia—to inactivation, but the anti-inflammatory property was correlated with C/EBP-α/PU.1 regulation. Hence, miR-124 alleviated vasoregression and improved the neuroretinal function.

In conclusion, our study demonstrates that miR-124 inhibits microglial activation and motility, promotes microglial polarization to the anti-inflammatory phenotype (M2 state), and thus decreases vasoregression and improves neuroretinal function in the PKD retina. Introduction of miR-124 as an early medical intervention to instruct microglia to shift to an anti-inflammatory phenotype is of great importance in maintaining retinal neurovascular function before entering advanced disease states.

## 4. Materials and Methods

### 4.1. Rat and Intravitreal Injection

The transgenic PKD rat model was generated at the Center of Medical Research of the Medical Faculty Mannheim at the University of Heidelberg [12]. The animals were held in a 12-h light and dark cycle with free access to food and water. The rats received intravitreal injections of 25 pmol of the miR-124 mimic (miR-124), control miRNA (CTL-miR), or miR-124-inhibitor (miR-inh) at week 8 and week 10 and were sacrificed at week 12. The eyes were enucleated and stored in 4% formalin or frozen at −80 °C. The wild-type SD rats were used as a control. The animal experiments were carried out in compliance with the statement of Association for Research in Vision and Ophthamology (ARVO) and approved by the local governmental authorities (animal license numbers G-150/16, Regierungspräsidium, Karlsruhe, Germany).

### 4.2. Retinal Digest Preparation and Quantitative Morphometry

The vascular preparation of rat eyes was obtained by trypsin digestion as described previously [53,54]. Briefly, the rat eyes were fixed in 4% formalin overnight. Then, the retina was separated from the eyecup. After brief washing with distilled water, the retina was digested in a 0.2 mol/L Tris-HCl (pH 7.4) buffer with 3% trypsin for 3 h. The extra tissues between capillaries were washed away with water drops. The retinal vascular net was dried on a glass slide and stained with Periodic Acid Schiff’s reagent (PAS) and hematoxylin.

Ten randomly selected areas were imaged with an Olympus BX51 Microscope under 400× magnification, and the retinal quantitative morphometry was carried out using an image analysis system of Cell^F^ (Olympus Opticals, Hamburg, Germany). Quantification of pericytes (PCs) or migrating pericytes (MPs) was calculated as the cell number relative to the retinal capillary area (cell number/mm^2^ capillary area), while quantification of the acellular capillaries was calculated as the number relative to the retinal area (AC number/mm^2^ retina area), according to the established methods and morphology definition described previously [55,56,57].

### 4.3. miRNA In Situ Hybridization (ISH) and Fluorecent Immunohistochemistry

The formalin-fixed and paraffin-embedded retinal samples were cut into 6-µm thick sections using SuperFrost Plus^TM^ (Thermo Fisher Scientific, Waltham, MA, USA) slides. The tissue sections were baked at 60 °C for 45 min, rehydrated in a gradient ethanol (100%, 95%, 80%, or 70%) each for 5 min, and briefly washed in a PBS buffer for 2 min. After denaturing the miRNA by heating at 90 °C for 4 min, the slides were treated with 20 µg/mL proteinase K at 37 °C for 10 min. The tissues were hybridized with an 80-nM Digoxigenin (DIG)-labeled miRCURY LNA miR-124 Detection Probe (Qiagen, Hilden, Germany) or scrambled miRNA probes (Qiagen) at 53 °C for 1 h. The slides were washed in 5 × SSC, 2 × SSC, and 0.2 × SSC buffers at 45 °C for 5 min. After an additional wash in a 0.2 × SSC solution at room temperature, the samples were blocked with 3% BSA containing 0.1% Tween-20 in PBS in a humidity chamber at room temperature for 20 min. Then, the tissues were incubated with sheep anti-digoxigenin (Roche, cat#11333089001, 1:800) and rabbit anti-glutamine synthetase (anti-GS) (Sigma, St. Louis, MO, USA, cat#G2781, 1:100) antibodies at 4 °C overnight. After being washed twice with PBS-T (0.1% Tween-20 in PBS), donkey anti-sheep Alexa Fluor 555 (Invitrogen, Waltham, MA, USA, cat#A21436, 1:200) and chicken anti-rabbit Alexa Fluor 488 (Invitrogen, cat#21441, 1:200) were added onto the samples at room temperature for 1 h, and the nuclei were stained with DRAQ5^TM^ (Invitrogen, cat#65-0880, 1:1000) for 10 min. The images were acquired with a Leica TCS SP8 confocal microscope (Leica Microsystems, Wetzlar, Germany) under constant exposure time and gain for all specimens, including negative scrambled miRNA controls.

### 4.4. Primary Culture of Rat Microglia

The primary microglial cells were isolated from the male homo-PKD rats at 3 months of age using magnetic beads from Neural Tissue Dissociation Kit-Postnatal Neurons (Miltenyi Biotec, cat#130-094-802, Bergisch Gladbach, Germany) according to the manufacturer’s instructions. The isolated retina was immersed in 6 mL of Dulbecco’s phosphate buffered saline (D-PBS) (Sigma, Taufkirchen/Munich, Germany) and then digested with Enzyme mix 1 (buffer with enzyme P) for 15 min at 37 °C and Enzyme mix 2 for 10 min. The retinal tissue was then dissociated by pipetting the whole solution up and down around 10 times. The cell suspension was loaded onto a MACS SmartStrainer (70 µm) (Miltenyi Biotec). After centrifugation at 1200 rpm for 5 min, the cells were collected and labeled with rabbit anti-CD74 antibody (Santa Cruz, cat#SC-20082, 1:100, Heidelberg, Germany) for 1 hour at room temperature. After sorting with anti-rabbit magnetic beads, the CD74^+^ cells were harvested and seeded in fibronectin-coated T25 flasks and cultured in a MACS Neuro medium (Miltenyi Biotec, cat#130-093-570) in a humidified incubator at 37 °C with 5% CO_2_.

### 4.5. Cell Line Cultivation and Transfection

The mouse microglia BV2 cell line was cultured in a DMEM medium containing 10% fetal bovine serum (FBS) (Invitrogen, cat#A4766801) supplemented with 100 U/mL penicillin and 100 µg/mL streptomycin (Invitrogen). In the 24-well cell culture format, 1 µL of 20 µM miRCURY LNA miR-124-3p mimic (Qiagen, cat#471256-001, sequence: 5′-UAAGGCACGCGGUGAAUGCC-3′), miRCURY LNA control miRNA mimic (Qiagen, cat#479903-001, sequence: 5′-GAUGGCAUUCGAUCAGUUCUA-3′), or miR-124 inhibitor (Qiagen cat#4102198-001, sequence 5′-GCATTCACCGCGTGCCTTA-3′) was transfected into the cells using a Lipofectamine 2000 Transfection Reagent (Invitrogen, cat#11668-027) according to the manufacturer’s instructions. Each miRNA mimic had a fluorescein labeled form (FAM), allowing observation after introduction.

### 4.6. Analysis of mRNA and miRNA Expression

The total RNA was extracted from the retinae with a TRIzol Reagent (Invitrogen, Waltham, MA, USA) according to the manufacturer’s instructions. For the messenger RNA (mRNA) quantification of the protein-encoding genes, 1 µg of RNA was reverse transcribed to first strand complementary DNA (cDNA) using a QuantiTect Reverse Transcription Kit (cat#205310, Qiagen, Hilden, Germany). The mRNA levels were determined using a real-time quantitative polymerase chain reaction (RT-qPCR) performed in a StepOne Plus Real-Time PCR system (Applied Biosystems, Waltham, MA, USA). Reactions were performed in a MicroAmp Optical 96-well Reaction Plate (Thermo Fisher Scientific, Dreieich, Germany) with the following steps: 50 °C for 2 min, 95 °C for 10 min, and then 40 cycles of 95 °C for 15 s and 60 °C for 1 min per cycle. Rat gene glyceraldehyde-3-phosphate dehydrogenase (Gapdh) was used as the internal control. The relative expression of each gene was normalized and calculated using the ΔΔCt method. The catalogue numbers of the primers are listed in Table 1.

Reverse transcription for the miRNA was performed using a miRCURY LNA RT-Kit (Qiagen, cat#339340) with 10 ng total RNA. Quantitative evaluation of miR-124 expression was detected using a miRCURY LNA SYBR PCR Kit (Qiagen, cat#339345) according to the manufacturer’s instructions. As the sequences of mature miR-124 in humans, mice, and rats are completely identical [58], we used the hsp-miR-124-3p Primer Set (Qiagen, cat#YP00206026) in this study. The expression of miRNA was normalized to U6 small nuclear RNA (snRNA).

### 4.7. Whole Mount Immunohistochemistry

After separation from the eyecup, the whole-mount retina was blocked and permeabilized in PBS with 1% BSA and 0.5% Triton X-100 for 2 h at room temperature. Following brief washing with PBS, the retina was incubated with biotin-conjugated iso-lectin B4 antibody (Sigma, cat#L1240, 1:100) and anti-CD74 antibody (Santa Cruz, cat#SC-20082, 1:100) at 4°C overnight. The secondary antibodies of porcine anti-rabbit TRITC (DAKO, Glostrup, Denmark, cat#R0156, 1:200) and streptavidin Alexa Fluor^®^ 633 (Invitrogen, cat#S21375, 1:500) were incubated at room temperature for 1 hour in the dark. All images were scanned using a Leica TCS SP8 confocal microscope. The microglia were defined as expressing CD74 and quantified in 10 randomly selected fields (400× magnification) from superficial and deep vascular retinal layers.

### 4.8. Immunocytochemistry (ICC)

After transfection for 24 h, the cells were re-seeded on the coverslips in a 24-well cell culture plate of 50–60% confluency. The cells were fixed with 4% formalin for 10 min and blocked in PBS with 1% BSA and 0.5% Triton X-100 for 30 min at room temperature. Then, the cells were incubated with primary antibodies against CCL2 (Millipore, Burlington, MA, USA, cat#MABN712, 1:100), CCL3 (Acris, cat#PP038P2, 1:100), PU.1 (Abcam, Cambridge, UK, cat#ab88082, 1:100), or Flot1 (Abcam, cat#ab41927, 1:100) at 4 °C overnight. Secondary antibodies, namely Alexa Fluor 555 donkey anti-mouse (Life technologies, Carlsbad, CA, USA, Ref: A31570) and Alexa Fluor 555 donkey anti-rabbit (Life technologies, Ref: A31572), were applied at a 1:200 dilution for 1 hour at room temperature in the dark. The nuclei were stained with DRAQ5^TM^ at a 1:1000 dilution for 30 min at room temperature. After washing with PBS three times, the slides were mounted with a fluorescence-preserving VECTASHIELD^®^ HardSetTM Antifade Mounting Medium (Vector Laboratories, cat#H-1400). The images were taken with a Leica TCS SP8 confocal microscope.

### 4.9. Western Blot

A frozen retina from a SD or PKD rat was homogenized in a 100-µL lysis buffer containing 0.1% SDS, 1% TritonX 100, 0.5% deoxycholate, 25 mmol/L HEPES pH 7.3, 10 mmol/L EDTA, 125 mmol/L NaCl, 10 mmol/L NaF, 10 mmol/L Na_4_P_2_P_2_, and 2 mmol/L of orthovanadate and protease inhibitor. After centrifugation at 14,000 rpm at 4 °C for 20 min, the supernatant was harvested. The protein concentration was determined with Bio-Rad Protein Assay Dye Reagent Concentrate (BIO-RAD, Hercules, CA, USA, cat#500-0006). Ten µg of protein was separated in 4–20% serial TGX^TM^ SDS-polyacrylamide gel (BIO-RAD, cat#456-1096). The proteins were transferred onto a 0.2-µm PVDF membrane using the Bio-Rad Trans-Blot Turbo Transfer System (BIO-RAD). The membrane was then blocked with 5% non-fat milk in PBS with 0.1% Tween 20 and incubated with primary antibodies at a 1:1000 dilution of anti-PU.1 antibody (Abcam, cat#ab88082) or anti-alpha Tubulin antibody (Abcam, cat#ab4074) at 4 °C overnight. After incubation with the Horseradish Peroxidase (HRP)-conjugated secondary antibody goat anti-mouse (DAKO, cat#P0447) and goat anti-rabbit (DAKO, cat#P0448) at a 1:2000 dilution at room temperature for 1 h, the immunoreactivity was visualized with Enhanced Chemiluminescence Substrate (Western Lighting ECL Pro, PerkinElmer). The Fusion SL system (Peqlab) was used for imaging and the densitometry was measured using Image J software.

### 4.10. Transwell Migration Assay

A total of 10^5^ BV2 cells were seeded in a Transwell^®^ (Biocompare, Germany) with an 8-µm pore size for the polyester membrane in the 24-well cell culture plate, and 100 µL of Opti-medium with a transfection reagent and the miRNA or control miRNA were added to the cells, with the downside of the well containing only 750 µL of Opti-medium. After 24 h of incubation at 37 °C, the cells still inside the Transwell membrane were removed with cotton sticks, and the cells that migrated to the lower side of the transwell were photographed and counted.

### 4.11. Wound Healing Assay

A scratch was made in the 24-well cell culture plate with confluent primary microglial cells, and the Opti-medium with a transfection complex (Lipofectamine 2000 addition with the miR-124 or control miRNA) was added. Images were taken at time points of 0 h and 24 h with a Leica DM IRB microscope, and at the end point, the cells were stained with Giemsa solution (cat#T862.1 ROTH, Berlin, Germany). The wound sizes were quantified with Image J software.

### 4.12. Live Cell Imaging and Time Lapse Analysis

After transfection for 24 h, the BV2 cells were reseeded in a new 24-well cell culture plate with a density of 4 × 10^4^ per well. Four hours later, the culture medium was changed to a serum-free medium, and live cell imaging was started with the Incucyte S3 system (Essen Biosciences). Phase contrast images were taken at 30-min intervals for 20 h, videos were exported in an uncompressed AVI format, and cell moving distances were analyzed by the Timelapse Analyzer program. The average migration distance (AMD) of each single cell was measured and expressed in micrometers. In all, 100 cells were analyzed per imaging field, and an average of 3 fields for each experimental condition was shown.

### 4.13. Electroretinography (ERG)

The neuroretinal function was measured by multifocal electroretinography under photopic conditions as previously described [59]. The RETImap system was utilized for the measurement (Roland Consult, Brandenburg an der Havel, Germany).

### 4.14. Statistical Analysis

Data are presented as means ± SD or means ± SEM. A Student’s t-test or one-way or two-way analysis of variance (ANOVA) with Tukey’s multiple comparisons test was used to determine the statistical differences between the experimental groups (Prism, GraphPad Software, San Diego, CA, USA), and a *p* value < 0.05 was considered statistically significant.

## Figures and Tables

**Table 1:** List of primers used in this study.

Gene Name	Reference Number *
Arg1	Rn00567522_m1
CCL2	Rn00580555_m1
CCL3	Rn01464736_g1
CD74	Rn00565062_m1
Cebp-α	Rn00560963_s1
IL-1ß	Rn00580432_m1
IL-10	Rn00563409_m1
IFN-γ	Rn00594078_m1
Gapdh	Rn99999916_s1
MHC-II	Rn01428452_m1
Spi1/PU.1	Rn01513815_m1
TGF-ß1	Rn00572010_m1
TNF-α	Rn01525859_g1

* All primers were from Thermo Fisher Scientific.

## Abbreviations

Arg1	Arginase-1
BMDMs	Bone marrow-derived macrophages
BSYS	Bu Shen Yi Sui capsule
CCL2	C-C motif chemokine ligand 2
CCL3	C-C motif chemokine ligand 3
CD74	Major histocompatibility complex, class II invariant chain
C/EBP-α	CCAAT/enhancer-binding protein-α
CNS	Central nervous system
DR	Diabetic retinopathy
EAE	Experimental autoimmune encephalomyelitis
ERG	Electroretinography GCL ganglion cell layer
Flot1	Flottilin-1
Gapdh	Glyceraldehyde 3-phosphate dehydrogenase
GS	Glutamine synthetase
IFN-γ	Interferon gamma
IL-1β	Interleukin-1β
IL-10	Interleukin-10
ILM	Inner limiting membrane
INL	Inner nuclear layer
IPL	Inner plexiform layer
MHC-II	Major histocompatibility complex class II molecule
miR-124	MicroRNA-124
miR-inh	MicroRNA-124 inhibitor
NVU	Neurovascular unit
OLM	Outer limiting membrane
ONL	Outer nuclear layer
OPL	Outer plexiform layer
PD	Photo-oxidative damage
PKD	Polycystic kidney disease
PU.1	Protein binding U box
SCI	Spinal cord injury
SD	Sprague Dawley
Spi1	Salmonella Pathogenicity Island 1 (gene name of PU.1)
TNF-α	Tumor necrosis factor-α
TNFR	TNF receptor
